# Phytase Overdose in Diets for Pigs from Weaning to Slaughter: Effects on Performance, Carcass and Meat Quality

**DOI:** 10.3390/vetsci13060516

**Published:** 2026-05-26

**Authors:** Cristina Satie Hideshima Marques, Marco Aurélio Callegari, Cleandro Pazinato Dias, Kelly Lais de Souza, Claudia Cassimira da Silva Martins, Vitor Barbosa Fascina, Alexandre Oba, Rafael Humberto de Carvalho, Caio Abércio da Silva

**Affiliations:** 1 Postgraduate Course of Animal Science, Center of Agrarian Science, State University of Londrina, Londrina 86057-970, Brazil; cristinahideshima@gmail.com (C.S.H.M.); oba@uel.br (A.O.); rafael.carvalho@uel.br (R.H.d.C.); 2 Akei Animal Research, São Paulo 18870-970, Brazil; marco.aurelio@akei.com.br (M.A.C.); cleandro@cleandrodias.com.br (C.P.D.); kelly.souza@akei.com.br (K.L.d.S.); 3 DSM Nutritional Products Ltd., São Paulo 05321-010, Brazil; claudia.silva@dsm-firmenich.com; 4 Novozymes Latin America Ltd., Araucária 83707-660, Brazil; vitfa@novonesis.com

**Keywords:** phytic acid, enzyme, phytate, minerals, myo-inositol

## Abstract

This study aimed to evaluate the extra-phosphoric effect of increasing doses of bacterial phytase (RONOZYME HiPhos) in corn- and soybean meal-based diets on performance, carcass yield, and meat quality in pigs during the nursery, growing, and finishing phases (GT). Two hundred and fifty pigs, castrated males and females, with an initial weight of 6.08 ± 0.748 kg and 21 days of age, were allocated to a randomized complete block design based on initial body weight, with five treatments and ten replicates per treatment: PC: positive control diets, supplemented with inorganic phosphorus (P) and calcium (Ca), meeting their full nutritional requirements; NC: negative control diets, with reduced available phosphorus (−0.18%) and calcium (−0.16%); 1000 FYT: NC + 1000 phytase units (FYT)/kg of feed; 2000 FYT: NC + 2000 FYT/kg of feed; 3000 FYT: NC + 3000 FYT/kg of feed. Average daily gain (ADG) in the nursery phase did not differ between the groups supplemented with 1000, 2000 and 3000 FYT/kg and PC, but was higher (*p* < 0.05) than NC. Feed conversion ratio (FCR) in the same phase was similar between PC and the groups supplemented with phytase, all being better (*p* < 0.05) than NC. The quadratic effect for phytase was verified for FCR in the phase, with the best inclusion of 2320 FYT/kg of feed. In the GF phases and in the overall experimental period (21 to 156 days), the results for average daily feed intake (ADFI), ADG and FCR favored PC and the groups supplemented with phytase compared to the NC (*p* < 0.05). A quadratic effect was observed for FCR considering the entire GF phase, with the best inclusion of 1923 FYT/kg of feed. Groups supplemented with phytase and PC obtained better carcass results compared to NC (*p* < 0.05). Linear effects were observed to percentage and quantity of lean meat in the carcass. There was no difference between treatments for meat quality. Supplementation with phytase in corn- and soybean meal-based diets with severely reduced inorganic P and Ca improved pig performance at all stages, with optimized inclusion values of approximately 2200 FYT/kg of feed, and dose-dependent benefits on carcass characteristics.

## Simple Summary

Phytase is the most important commercial enzyme used in swine feed. The concept of phytase overdosing in swine is still limited, with several positive but varied scientific findings. This study evaluated the effect of phytase overdosing [1000, 2000, and 3000 phytase units (FYT)/kg of feed] in corn- and soybean meal-based diets with severely reduced levels of inorganic phosphorus and calcium (−0.18% available phosphorus and −0.16% calcium), compared to adequately supplemented diets deficient in these minerals, in all phases from weaning to commercial slaughter age, considering performance, carcass characteristics, and meat quality. Phytase supplementation improved swine performance in all phases, with optimized inclusion values of approximately 2200 FYT/kg of feed, and dose-dependent benefits in carcass characteristics, but did not influence meat parameters.

## 1. Introduction

In animal production, dietary phosphorus (P) is derived primarily from non-renewable sources, with a smaller proportion originating from plant-based ingredients in feed. Therefore, its efficient utilization is required, and its bioavailability must be accurately characterized [1]. The precise determination of the P requirements, in addition to aligning dietary supply with the nutritional demands of animals, also contributes to mitigating negative environmental impacts [1,2].

P is the second most abundant mineral in the body, after calcium (Ca) [3,4], and is an essential macroelement that must be supplemented in pig diets [5]. In plant-based feed ingredients, P is predominantly present as phytate, accounting for up to 80% of total P, which has limited availability for monogastric animals [6], due to the absence of endogenous phytase activity.

According to Lautrou et al. [1], approximately 60% of the P in the animal body is located in bone in a fixed proportion with calcium, while the remainder is distributed in soft tissues, primarily muscle. Thus, meeting the requirements for these minerals must be considered jointly, since P utilization is closely related to Ca absorption and metabolism. Furthermore, the formation of insoluble and indigestible Ca–P complexes in the intestine may impair mineral availability [7].

To improve the digestive and metabolic utilization of P, and consequently reduce its excretion, phytases, enzymes that increase the availability of plant-derived P, present as phytic acid, a phosphoric ester of inositol, are widely used as feed additives in pig and poultry diets. Phytases are currently the most extensively applied enzymes in animal nutrition [8], and their effects are typically dose-dependent up to a certain inclusion level [9,10].

Within this dose–response framework, supplementation with phytase at levels exceeding those required to release phytate-bound P is defined as phytase superdosing, generally involving inclusion rates above 500 FTU/kg and up to 2500 FTU/kg [11,12]. Studies in pigs have demonstrated that phytase superdosing can improve growth performance compared to conventional inclusion levels used in commercial diets [10,13,14,15]. Furthermore, phytase supplementation at high inclusion levels also reduces phosphorus excretion in feces and urine in a dose-dependent manner, as reported by Czech et al. [16], who evaluated diets with reduced Ca and P content (approximately 75 to 85% lower than the control diet) supplemented with 250 to 1500 FTU/kg feed for growing–finishing pigs.

Another attractive point of phytase superdosing is its potential to improve the economic viability of pig production by enhancing growth performance and productive efficiency while reducing feed costs, as reported by Yuanfeng et al. [13] and more recently confirmed by Zhao et al. [17], who evaluated Ca- and P-deficient diets supplemented with 500 to 3000 FTU/kg feed for growing–finishing pigs.

The extra-phosphoric effects associated with phytase superdosing are attributed to the release of myo-inositol and the more complete and rapid degradation of antinutritional inositol phosphate esters [12]. Myo-inositol plays a critical role in cellular processes, functioning as a component of phospholipids and inositol phosphates, and is essential for a wide range of biological functions, including cell growth and survival, peripheral nerve development and function, and osteogenesis. Additionally, it has been associated with increased insulin sensitivity and reductions in total cholesterol and triglyceride levels. In reproduction, it restores ovulatory activity, improving oocyte quality, as well as sperm motility and membrane potential, and in neurological processes, it influences serotonin levels [18].

Although the use of phytase at levels exceeding traditional recommendations represents a relatively recent strategy in swine nutrition, further investigation is still required to fully elucidate its effects [13]. Therefore, the present study provides a comprehensive evaluation of phytase superdosing in corn- and soybean meal-based diets, assessing its impact on growth performance, carcass characteristics, and meat quality of pigs over an extended production period, from weaning to commercial slaughter age, under conditions of more severe dietary calcium and phosphorus restriction than those commonly reported in the literature [10,14,19,20,21]. This approach may also contribute to improving the economic viability of growing–finishing pig production and reducing environmental impacts associated with fecal and urinary P excretion.

## 2. Materials and Methods

The study was conducted in accordance with the recommendations of the Guide for the Care and Use of Laboratory Animals of the National Council for the Control of Animal Experimentation (CEUA) and was approved by the Ethics Committee on Animal Experimentation of Akei Animal Research (protocol number: 004/21).

### 2.1. Animals and Housing

A total of 250 commercial PIC (AG337 × Camborough) piglets were used, including 125 females and 125 barrows, weaned at approximately 21 days of age, with an average body weight of 6.079 ± 0.748 kg. Pigs were housed in pens, with five animals of the same sex per pen.

Each pen had an area of 5.5 m^2^ and was equipped with a nipple drinker and a Dutch-type feeder. Thermal control was performed manually by adjusting side curtains in the barn. Air temperature and relative humidity were recorded throughout the experimental period using a data logger (Instrutemp ITLOG 80, São Paulo, Brazil).

### 2.2. Experimental Treatments and Diets

The experimental design was a randomized complete block design based on initial body weight at 21 days of age, with five treatments, ten replicates, and five animals per pen. The treatments were as follows: PC, positive control diets supplemented with inorganic phosphorus and calcium to meet the nutritional requirements; NC, negative control diets with reduced available phosphorus (−0.18%) and calcium (−0.16%); 1000 FYT, NC supplemented with 1000 phytase units (FYT)/kg of feed; 2000 FYT, NC supplemented with 2000 FYT/kg of feed; and 3000 FYT, NC supplemented with 3000 FYT/kg of feed. RONOZYME HiPhos (DSM Nutritional Products, São Paulo, Brazil) was used as the phytase source, consisting of a 6-phytase produced by introducing synthetic gene sequences that mimic a phytase gene from *C. braakii* ATCC 51113 and expressed in *A. oryzae* [22].

The animals were subjected to a feeding program consisting of eight phases: pre-starter I (21 to 28 days of age), pre-starter II (29 to 35 days of age), starter I (36 to 49 days of age), starter II (50 to 63 days of age), growth I (64 to 91 days of age), growth II (92 to 112 days of age), finish I (113 to 133 days of age), and finish II (134 to 156 days of age) (Table 1 and Table 2). All diets, based on corn and soybean meal, were formulated to meet the minimum nutritional requirements according to the Brazilian Tables for Poultry and Swine [23] except for calcium and phosphorus levels in the NC diets. Feed and water were provided ad libitum throughout the experimental period.

### 2.3. Performance and Carcass Analyses

Average daily feed intake (ADFI), average daily gain (ADG), and feed conversion ratio (FCR) were evaluated at the beginning of the study and at the end of each experimental phase, corresponding to 28, 35, 49, 63, 91, 112, 133, and 156 days of age. At 156 days of age, all animals were slaughtered after a 12 h fasting period before transport. Animals were stunned by electronarcosis and subsequently slaughtered by sectioning the neck vessels.

The carcasses were subjected to electronic classification using a Hennessy Grade Probe (Hennessy Grading Systems, Auckland, New Zealand) by measuring backfat thickness (BT) and Longissimus thoracis et lumborum muscle depth (LD) at point P2, located 59 mm lateral to the dorsal midline of the carcass, immediately caudal to the last rib on the left half-carcass [24].

The carcasses were weighed to determine carcass weight (CW), and the percentage and content of lean meat (LM) in the carcass were obtained. Lean meat percentage was calculated based on the modified equation proposed by Hennessy Grading Systems: % LM = 61.33 − (0.76 × BT) + (0.1 × LD). Lean meat content was calculated by multiplying carcass weight by lean meat percentage.

### 2.4. Meat Quality Assessments

After carcass chilling, 24 h postmortem, 45 carcasses were randomly selected, with 15 samples per treatment. A sample of the Longissimus dorsi muscle, located between the last and penultimate ribs of the left half-carcass, was collected for meat quality and lipid oxidation analyses. The final muscle pH was measured using a Hanna potentiometer. Meat marbling was determined using photographic standards and numerical scoring scales based on the American Meat Science Association guideline [25].

Color was determined after 30 min of sample exposure to oxygen using a CR-10^®^ portable colorimeter (Konica Minolta, Inc., Osaka, Japan) with illuminant D65, a 10° viewing angle, and an 8.0 mm aperture. The L (lightness), a* (redness-greenness), and b* (yellowness-blueness) components were evaluated using the CIELAB system [26].

Water-holding capacity (WHC) was measured using the pressure-induced water loss method and expressed as the percentage of exudate lost relative to the initial sample weight [27].

Meat tenderness was determined according to the methodology proposed by [28]. Samples were subjected to shear force analysis using a Warner-Bratzler blade coupled to a Texture Analyzer TA-XT2i (Stable Micro Systems Ltd., Godalming, Surrey, UK).

Lipid oxidation was evaluated on the day of freezing and after seven days after thawing. Lipid oxidation was determined using the 2-thiobarbituric acid reactive substances (TBARS) assay, adapted from Tarladgis [29] and modified by [30].

### 2.5. Statistical Analysis

Data were subjected to analysis of variance (ANOVA) using the General Linear Model (GLM) procedure and regression analysis in SAS (Statistical Analysis System, version 9.4, Cary, NC, USA). Means were compared using Tukey’s test. Linear, quadratic, and cubic regression analyses were performed to evaluate the dose–response effects of phytase supplementation. Regression graphs were generated using GraphPad Prism version 9.5. The pen was considered the experimental unit for performance parameters, and the individual animal was considered the experimental unit for carcass traits and meat quality parameters. The significance level for differences between means and regression effects was set at α = 0.05.

## 3. Results

In the pre-starter phase, no differences (*p* > 0.05) were observed among treatments for any parameter (Table 3). In pre-starter phase II (29 to 35 days of age), a difference (*p* < 0.05) was observed for FCR, in which the 1000 FYT, 2000 FYT, and 3000 FYT treatments showed improvements of 11.21%, 10.17%, and 10.38%, respectively, compared to NC, while PC did not differ from the other treatments. For ADG, ADFI, and final weight (FW), no differences were detected among treatments. However, a quadratic effect was observed for FCR, with the optimal inclusion estimated at 2050 FYT/kg of feed (Table 3). In initial phase I (36 to 49 days of age) (Table 3), PC showed higher ADG (*p* < 0.05) compared to NC and 1000 FYT, with increases of 25.35% and 16.15%, respectively, while 2000 FYT and 3000 FYT did not differ from the other treatments. The FCR of NC animals was higher (*p* < 0.01), indicating poorer efficiency compared to the other treatments. A quadratic effect was observed, with optimal inclusions of 2211 FYT/kg of feed for ADG and 2220 FYT/kg of feed for FCR in this phase.

In initial phase II (50 to 63 days of age), a difference (*p* < 0.05) was observed in final weight between PC and NC, with a 13.36% advantage for PC (Table 3). Considering the entire nursery phase (Table 3), PC showed the highest ADG compared to NC, with an increase of 17.25%, while the other treatments did not differ from PC. FCR was higher in the NC group, indicating poorer efficiency, with differences of 9.36%, 7.75%, 9.48%, and 8.41% compared to PC, 1000 FYT, 2000 FYT, and 3000 FYT, respectively. A regression effect was observed for FCR, with the optimal dose estimated at 2327 FYT/kg of feed, as illustrated by the regression-based dose–response curves for selected nursery performance responses (Supplementary Figure S1).

In the growth and finishing phases (Table 4), initial weight (IW) was included as a covariate. ADFI during the growth phase was 17.22% higher for PC (*p* < 0.05) compared to NC, while no differences were observed among the other treatments. ADG, FCR, and FW did not differ among PC, 1000 FYT, 2000 FYT, and 3000 FYT (*p* > 0.05), but all were superior to NC (*p* < 0.05). Quadratic effects (*p* < 0.05) were observed for ADG, FCR, and FW, with optimal inclusions of 2424, 2201, and 2133 FYT/kg of feed, respectively, as illustrated by the regression-based dose–response curves for performance responses during Growing I (Supplementary Figure S2).

In growth phase II (92 to 112 days of age), finishing phase I (113 to 133 days of age), and finishing phase II (134 to 156 days of age), PC, 1000 FYT, 2000 FYT, and 3000 FYT showed similar ADFI, ADG, and FW (*p* > 0.05), and all were superior to NC (Table 4). In growth phase II, quadratic effects were observed for ADFI, FCR, and FW, with optimal inclusions of 2457, 2257, and 2392 FYT/kg of feed, respectively. ADG showed a linear response to increasing phytase inclusion (Y = 0.8768 + 0.00074305X), with these regression-based dose–response effects shown in Supplementary Figure S3.

For finishing phase I, the regression-based dose–response effects of phytase supplementation on performance responses are shown in Supplementary Figure S4. In finishing phase II, a linear effect was observed for ADG and a quadratic effect for FCR, with the optimal inclusion estimated at 2687 FYT/kg of feed, as shown in Supplementary Figure S5. Considering the entire growth and finishing period (63 to 156 days of age), no differences were observed among PC, 1000 FYT, 2000 FYT, and 3000 FYT for any evaluated parameter (*p* > 0.05), whereas all treatments were superior to NC (*p* < 0.05). A quadratic effect was observed for FCR, with the optimal dose estimated at 1923 FYT/kg of feed, as further illustrated by the regression-based dose–response effects during the combined growing–finishing period (Supplementary Figure S6).

Throughout the entire experimental period (21 to 156 days) (Table 5), phytase supplementation, regardless of inclusion level, resulted in performance equivalent to PC (*p* > 0.05), and all treatments were superior to NC (*p* < 0.05). Positive linear effects were observed for ADG and ADFI, and a quadratic effect was observed for FCR, with the optimal phytase inclusion estimated at 2102 FYT/kg of feed, as illustrated by the regression-based dose–response effects on total-period performance shown in Supplementary Figure S7.

FW at slaughter, carcass weight (CW), carcass yield (CY), loin depth (LD), and lean meat content (LM) were similar among PC, 1000 FYT, 2000 FYT, and 3000 FYT (*p* > 0.05) and all were superior to NC (*p* < 0.05) (Table 6). The percentage of lean meat in the carcass (PLM) for PC, 2000 FYT, and 3000 FYT was higher than NC (*p* < 0.05), corresponding to increases of 4.27%, 3.92%, and 3.95%, respectively, while 1000 FYT did not differ from the other treatments. For backfat thickness (BT), no differences were observed among treatments (*p* > 0.05). Quadratic effects were observed for LD, with the optimal inclusion estimated at 2195 FYT/kg of feed. For the remaining characteristics, except BT, a positive linear effect was observed with increasing phytase levels (Table 6). These regression-based dose–response effects are illustrated for final weight, carcass weight, and carcass yield in Supplementary Figure S8, and for loin depth and carcass lean meat deposition in Supplementary Figure S9.

For meat quality (Table 7), no differences (*p* > 0.05) were observed among treatments for any evaluated parameter, and no linear or quadratic effects were detected.

## 4. Discussion

In the nursery phase, the ADG and FCR observed in phytase-supplemented groups, which were similar to PC (positive control), support the efficacy of phytase under superdosing conditions, whose effects are associated with enhanced phytate dephosphorylation and increased release of myo-inositol. According to Moran et al. [31], myo-inositol may act as a conditionally essential nutrient for piglets under weaning stress. In that study, two phytase levels (0 and 2500 FTU/kg) and three inositol concentrations (0%, 0.15%, and 0.30%) were evaluated, and phytase superdosing tended to improve ADG compared to diets without phytase. Similarly, increasing inositol concentrations improved feed efficiency in pigs fed diets without phytase, but no additional benefits were observed when inositol was combined with phytase supplementation. In the present study, exogenous inositol improved feed efficiency during the first ten days of the nursery period at levels comparable to phytase superdosing, which is consistent with the improvements observed during the second week post-weaning.

The use of increasing phytase levels (500, 1000, or 2000 FTU/kg of feed) in diets with reduced Ca and P for weaned pigs has been shown to reduce fecal excretion of these minerals and increase their retention, resulting in improved performance [6]. The present results are consistent with these findings, as treatments supplemented with phytase (1000 FYT, 2000 FYT, and 3000 FYT) showed performance similar to PC and superior to NC from the pre-starter II phase onward, indicating that phytase superdosing during the nursery phase is effective and that its benefits may become more evident during the growth and finishing phases.

In the present study, the reduction of approximately −0.18% and −0.16% in dietary P and Ca, respectively, in phytase-supplemented diets contrasts with other studies that reported less pronounced reductions in these minerals [10,14,20]. However, regardless of the phytase inclusion level, performance was similar to PC (Table 3), which contained adequate mineral levels according to established nutritional requirements, typically higher during the nursery phase than in later production stages [23]. These results suggest that phytase supplementation was sufficient to compensate for the reduced mineral levels, maintaining performance comparable to PC. The absence of differences between phytase treatments and NC for some variables in the nursery phase may be related to the relatively high mineral requirements at this stage, whereas the improved responses observed during later phases may reflect the lower mineral demands of older animals.

Regarding the optimal phytase inclusion levels, the best performance responses were observed at higher doses, consistent with findings reported by [15], in nursery pigs. In the present study, regression analyses indicated optimal inclusion levels ranging from approximately 2058 to 2327 FYT/kg of feed for ADG and FCR, respectively.

The improvements observed with phytase supplementation during the nursery phase may be explained by the greater sensitivity of young piglets to dietary interventions, particularly due to reduced endogenous enzyme activity and the abrupt dietary transition at weaning. Consequently, even modest improvements in nutrient digestibility, including starch and protein, may result in measurable performance gains, given the limited digestive capacity of newly weaned piglets [32].

Differences among treatments, particularly in comparison with NC, became evident during the nursery phase, initially in pre-starter I for FCR and more markedly during starter I. These responses may be attributed to the role of phytase in reducing the antinutritional effects of phytate, increasing the availability of nutrients, and promoting the release of myo-inositol, which may act synergistically to improve performance [32].

Phytate interacts with gastrointestinal enzymes such as α-amylase, and phytase superdosing may reduce this interaction, potentially increasing starch digestibility and, consequently, dietary energy utilization and animal performance. However, because most starch digestion occurs in the distal small intestine, the magnitude of this improvement may be limited in piglets, particularly during the post-weaning period, although still relevant due to their enzymatic immaturity and reduced digestive capacity [32].

In the growth and finishing phases, the present results are consistent with previous studies demonstrating the benefits of phytase superdosing [19], diets with greater reductions in P and Ca than those used in the present study, supplemented with increasing phytase levels (Ronozyme HiPhos, 250, 500, 1000, or 1500 FYT/kg of feed), resulted in lower ADG and poorer FCR in pigs fed the negative control diet compared to the positive control. In contrast, ADG improved with increasing phytase inclusion in P- and Ca-deficient diets during the finishing phase, and FCR was similar between phytase-supplemented treatments and PC.

According to [12], phytase supplementation above 500 FYT/kg of feed, characterizing superdosing, can improve pig performance and feed efficiency due to enhanced phytate hydrolysis and improved nutrient utilization.

In a study evaluating diets adequately supplemented with inorganic P (PC), P-deficient diets with reduced lysine and energy (NC), and NC supplemented with 2500 FTU/kg phytase across nursery, growing, and finishing phases, reported that PC resulted in higher final body weight, greater growth rate, and improved feed and energy efficiency compared to NC. Phytase superdosing improved performance, particularly during the growing and finishing phases, although effects were less pronounced during the nursery phase. The present findings are consistent with these observations, indicating phase-dependent responses in performance. It is likely that, in addition to increased P availability, phytase superdosing enhanced the availability of energy and amino acids, although the higher nutritional demands of nursery pigs may have limited the magnitude of the response at this stage.

In line with these observations, Varley et al. [33] reported that phytase supplementation in nursery diets deficient in P, followed by adequate P supply during the growing and finishing phases, resulted in improved performance in subsequent phases.

Similarly, Santos et al. [20] observed that phytase supplementation improved ADG and FCR in growing and finishing pigs fed P- and Ca-deficient diets, despite using smaller reductions in these minerals (0.16% and 0.15%, respectively) and only two phytase levels (500 and 2500 FYT/kg of feed).

In another study with castrated male pigs fed diets with low or adequate phytase content and supplemented with 250, 500, 2500, or 12,500 FYT/kg for 14 days, Tsai et al. [21] reported no effect on ADFI, but an improvement in ADG at 2500 FYT/kg. Consistent with these findings, the present study demonstrates that phytase superdosing improves performance under conditions of more pronounced reductions in dietary Ca and P (−0.16% and −0.18%, respectively), which are greater than those reported in previous studies [10,14,19,20,21]. These results reinforce the applicability of phytase superdosing even under more severe mineral restriction conditions.

The responses to increasing phytase levels on performance during the growth and finishing phases were generally linear, consistent with dose-dependent effects [33] or exhibited quadratic responses with optimal levels ranging from 2000 to 2400 FYT/kg of feed, in agreement with the findings of [10,21]. However, these results extend previous observations, as the dietary Ca and P reductions applied in the present study were greater than those reported in the cited studies.

Although studies applying more severe dietary Ca and P deficits are limited and generally older, the results obtained in the nursery phase in the present study were superior to those reported by [34], who used diets supplemented with 2000 FTU/kg feed during the same phase, with deficits of 0.18% P and 0.25% Ca relative to the control diet. In our study, the FCR of pigs supplemented with phytase (with an estimated optimal dose of 2327 FYT/kg feed) was 8.41% lower than that of the NC group, compared with a 6.85% reduction reported in the cited study, highlighting the efficacy of the enzyme under the evaluated dietary restriction conditions.

For the growing-finishing phases [35], using diets supplemented with 10,000 FTU/kg feed and P restrictions comparable to the greatest P reduction applied in the present study, observed improvements in ADG and FCR of 10.0% and 9.21%, respectively, compared with the unsupplemented group. In the present study, the improvement in ADG was greater, reaching 24.66% compared with the NC group, whereas the improvement in FCR was similar, at 9.35% compared with NC. These findings reinforce the effectiveness of the phytase evaluated here in mitigating performance losses caused by dietary mineral deficiency. The benefits of phytase observed in this study are largely associated with improvements in the digestibility of P and Ca. In this context, Brady et al. [36] emphasized this effect, and the authors of [37], working with growing and finishing pigs, demonstrated that microbial phytase, even in diets with low total phosphorus, increased the apparent fecal digestibility of P and other nutrients, resulting in improved performance and reduced environmental excretion.

In a study with six treatments (PC, NC, and diets supplemented with 500, 1000, 2000, and 4000 FYT/kg of feed), Almeida et al. [38] reported improvements in Ca and P digestibility, with P digestibility being approximately threefold higher than that of Ca. Evaluating the efficacy of Buttiauxella 6-phytase expressed in Trichoderma reesei, supplemented at 0, 500, 1000, or 2000 FTU/kg of feed [39] demonstrated that, in growing pigs, the enzyme increased the utilization of P and Ca and enhanced ileal digestibility of protein and several amino acids in a dose-dependent manner. These findings support the role of phytase and are consistent with the results observed in the present study.

The extra-phosphoric effects of phytase are closely associated with the concept of phytase superdosing, in which inclusion levels above conventional recommendations may enhance growth performance beyond responses expected solely from P release, particularly when adequate dietary phosphorus levels are not supplied [40,41]. This response is mainly attributed to greater phytate degradation, which markedly reduces its antinutritional effects. Phytate can form stable binary complexes through electrostatic interactions with free amino groups of proteins and with minerals such as calcium, zinc, and iron in the digestive tract, thereby compromising their absorption [11,42]. Consequently, the benefits of high phytase inclusion may also involve improvements in energy, amino acid, and mineral digestibility, beyond Ca and P utilization [43,44]. In parallel, the more extensive hydrolysis of the phytate molecule promoted by higher phytase inclusion can increase myo-inositol release, a compound involved in several metabolic functions [18]. Based on the studies presented, two main explanations may account for the observed results. First, the greater reductions in dietary Ca and P in the NC treatment likely contributed to its poorer performance compared to the other treatments. This finding is consistent with [10], who reported that phytase supplementation (1000, 2000, or 3000 FYT/kg of feed) in corn- and soybean meal-based diets with reduced inorganic phosphorus (−0.11%) and calcium (−0.13%) improved feed intake and ADG, with the lowest performance observed in pigs fed the NC diet. However, responses to phytase supplementation above 3000 FYT/kg appear to be limited, as reported by [14] who observed that positive responses were generally restricted to inclusion levels up to 3000 FYT/kg in similar diets. In the present study, a comparable pattern was observed, with linear responses for ADFI and ADG and quadratic responses for FCR across the entire growth and finishing period.

A second explanation may be related to the effect of phytase on restoring the available Ca:P ratio, primarily through a greater release of P relative to Ca. Thus, the improvement in performance may be attributed to enhanced utilization of plant-derived P and increased availability of nutrients such as starch and protein previously bound to phytic acid [36].

The absence of differences in ADG among phytase treatments during the finishing phase may be associated with the animals approaching their genetic potential for growth, thereby limiting further performance responses. Alternatively, endogenous phytase activity may increase with age, as the concentration of phytase in the intestinal mucosa rises, improving the capacity of older pigs to utilize dietary phytate [37].

Regarding carcass characteristics (Table 6), the present results differ from those reported by [6], Regarding carcass characteristics (Table 6), the present results differ from those reported by Dersjant-Li et al. [45]. Similarly, Fandrejewski et al. [46], using 1000 FYT/kg of phytase expressed in Aspergillus niger, reported no differences in carcass traits, which contrasts with the present findings obtained under higher phytase inclusion levels.

However, Lozano et al. [47], working with phytase expressed in Aspergillus niger in finishing pigs, reported greater loin depth with 500 and 1000 FYT/kg of feed compared to the negative control group. Some authors have reported increases in carcass fat percentage, attributing these effects to enhanced dietary energy utilization resulting from higher phytase inclusion levels. It should be noted that phytase activity is substrate-dependent, and in that study, diets included defatted corn germ meal, which differs substantially from corn in terms of phytate concentration.

Differences in the magnitude of phytase responses have been discussed by Kerr et al. [48], who compared different phytase sources and their capacity to release energy from diets. In this context, Brady et al. [36], using a phytase expressed in Peniophora lycii at inclusion levels of 500, 750, and 1000 FYT/kg of feed, observed a linear increase in backfat thickness and a linear reduction in lean meat content, indicating variability in responses depending on enzyme characteristics.

The present results are consistent with those reported by Lozano et al. [47], who observed increased loin depth with phytase supplementation, and with [10], who reported that phytase inclusion did not increase fat deposition, suggesting that the additional energy released by phytase may not be sufficient to promote lipid accretion, even when improvements in ADG and ADFI are observed.

Considering the effect of phytase level on carcass traits, the positive linear responses observed for final weight, carcass weight, carcass yield, and both the percentage and amount of lean meat are consistent with the dose-dependent action of the enzyme. The quadratic effect observed exclusively for loin depth, with an optimal inclusion of 2195 FYT/kg of feed, is comparable to optimal values identified for some performance parameters during the nursery phase, although this response has not been consistently reported in studies evaluating similar phytase inclusion ranges [10]. These findings suggest that the additional energy released by phytase was not sufficient to increase fat deposition, while also highlighting that variations in phytase response may be influenced by factors such as enzyme origin and expression system, inclusion level, and dietary composition.

Regarding meat quality, the present findings corroborate those of Lozano et al. [47], who reported no differences among treatments for all evaluated parameters, regardless of phytase inclusion level, as well as those of de Souza et al. [49], who observed no effects on color, pH, firmness, or shear force between phytase-supplemented and non-supplemented diets.

However, the present results are also consistent with Gebert et al. [50], who, using very high phytase inclusion levels (5000 FTU/kg), reported effects on meat color, with L values approximately 3% higher, indicating paler meat*. In that study, phytase supplementation did not significantly affect water-holding capacity or intramuscular fat content of the Longissimus dorsi muscle, but increased lipid oxidation (TBARS) was observed in the phytase-supplemented group.

## 5. Conclusions

Supplementation of corn- and soybean meal-based diets with reduced inorganic phosphorus and calcium, using 1000, 2000, or 3000 FYT/kg of phytase, improved feed intake and weight gain, and resulted in feed conversion ratios comparable to diets adequately supplemented with these minerals. These results demonstrate positive dose-dependent effects on growth performance across nursery, growing, and finishing phases, without affecting meat quality. Phytase inclusion levels ranging from approximately 1900 to 2300 FYT/kg feed were associated with optimal feed conversion responses across the experimental phases and over the entire study period under the conditions of the present study.

## Figures and Tables

**Table 1:** Composition and nutritional values of experimental diets for pre-starter I (21–28 days of age), pre-starter II (29–35 days of age), starter I (36–49 days of age), and starter II (50–63 days of age).

Ingredients (kg)	Rations							
	Pre-Starter I		Pre-Starter II		Starter I		Starter II	
	PC	NC	PC	NC	PC	NC	PC	NC
Corn grain (8.8%)	538.19	551.44	561.69	574.31	596.13	609.39	645.65	658.85
Soybean meal (45%)	206.00	206.00	248.00	248.00	280.00	280.00	289.00	289.00
Whey powder	150.00	150.00	100.00	100.00	50.00	50.00	0.00	0.00
Plasma Powder	50.00	50.00	25.00	25.00	0.00	0.00	0.00	0.00
Soybean Oil	16.70	11.30	22.00	16.80	26.20	20.76	23.80	18.40
Dicalcium Phosphate (24/18%)	19.25	9.20	18.57	9.62	19.60	9.53	18.45	8.40
Limestone Calcium (36%)	2.80	5.00	4.10	5.63	4.37	6.62	4.68	6.93
Common Salt	1.90	1.90	4.50	4.50	5.85	5.85	5.80	5.80
Zinc Oxide (72%)	3.50	3.50	3.50	3.50	3.50	3.50	0.00	0.00
Copper Sulfate Pentahydrate	0.50	0.50	0.50	0.50	0.50	0.50	0.50	0.50
DL Methionine	1.70	1.70	1.75	1.75	1.89	1.89	1.67	1.67
L Lysine HCl	4.45	4.45	4.91	4.91	5.66	5.66	5.18	5.18
L Threonine	2.00	2.00	2.25	2.25	2.66	2.66	2.13	2.13
L Tryptophan	0.44	0.44	0.47	0.47	0.55	0.55	0.45	0.45
L Valine	0.82	0.82	1.01	1.01	1.34	1.34	0.94	0.94
Mycotoxin Adsorbent ^1^	1.50	1.50	1.50	1.50	1.50	1.50	1.50	1.50
Mineral Premix ^2^	0.10	0.10	0.10	0.10	0.10	0.10	0.10	0.10
Vitamin Premix ^3^	0.15	0.15	0.15	0.15	0.15	0.15	0.15	0.15
Calculated Values								
ME, kcal/kg	3350	3350	3350	3350	3350	3350	3350	3350
Crude protein, %	20.06	20.17	19.89	19.99	19.42	19.53	19.51	19.64
SID Lysine, %	1.40	1.40	1.35	1.35	1.30	1.30	1.25	1.25
Calcium, %	0.75	0.59	0.75	0.59	0.75	0.59	0.7	0.54
Total phosphorus, %	0.71	0.54	0.68	0.52	0.68	0.5	0.64	0.46
Available phosphorus, %	0.55	0.37	0.5	0.34	0.48	0.3	0.43	0.25
Calcium: available phosphorus ratio	1.36	1.59	1.5	1.73	1.56	1.96	1.63	2.16
Sodium %	0.35	0.35	0.35	0.35	0.3	0.3	0.25	0.25
SID Met + Cys/SID Lys	0.56	0.56	0.56	0.56	0.56	0.56	0.57	0.57
SID Thr/SID Lys	0.67	0.67	0.67	0.67	0.67	0.67	0.65	0.65
SID Trip/SID Lys	0.19	0.19	0.19	0.19	0.19	0.19	0.19	0.19
SID Val/SID Lys	0.69	0.69	0.69	0.69	0.69	0.69	0.69	0.69
SID Ile/SID Lys	0.50	0.50	0.52	0.52	0.53	0.53	0.55	0.55

PC: Positive Control; NC: Negative Control; ME: metabolizable energy; SID = standardized ileal digestibility; ^1^ Mycotoxin adsorbent; ^2^ Mineral premix provided per kg of diet: 100 mg Fe; 10 mg Cu; 40 mg Mn; 1 mg Co; 100 mg Zn; 1.5 mg I. ^3^ Vitamin premix provided per kg of diet: 6000 IU vitamin A; 1500 IU vitamin D_3_; 15 mg vitamin E; 1.5 mg vitamin K_3_; 1.35 mg vitamin B_1_; 4 mg vitamin B_2_; 2 mg vitamin B_6_; 20 μg vitamin B_12_; 20 mg niacin; 9.35 mg pantothenic acid; 600 μg folic acid; 80 μg biotin; 300 μg Se.

**Table 2:** Composition and nutritional values of experimental diets for growing I (64–91 days of age), growing I (92–112 days of age), finishing I (113–133 days of age) and finishing II (134–156 days of age).

Ingredients (kg)	Rations							
	Growing I		Growing II		Finishing I		Finishing II	
	PC	NC	PC	NC	PC	NC	PC	NC
Corn grain (8.8%)	706.55	719.45	748.22	761.22	797.63	810.89	838.73	851.43
Soybean meal (45%)	235.00	235.00	199.00	199.00	161.00	161.00	125.00	125.00
Soybean oil	20.00	15.00	17.50	12.20	9.40	4.00	7.00	1.75
Dicalcium phosphate (24/18%)	14.80	4.70	12.10	2.10	10.58	0.52	9.05	0.00
Calcium limestone (36%)	7.50	9.70	8.00	10.30	7.90	10.10	7.70	9.30
Common salt	4.60	4.60	4.60	4.60	3.80	3.80	3.80	3.80
Copper sulfate pentahydrate	0.50	0.50	0.50	0.50	0.50	0.50	0.50	0.50
DL-Methionine	1.43	1.43	1.11	1.11	0.86	0.86	0.52	0.52
L-Lysine HCl	4.82	4.82	4.58	4.58	4.35	4.35	4.11	4.11
L-Threonine	1.82	1.82	1.61	1.61	1.40	1.40	1.20	1.20
L-Tryptophan	0.54	0.54	0.51	0.51	0.48	0.48	0.46	0.46
L-Valine	0.69	0.69	0.52	0.52	0.35	0.35	0.18	0.18
Mycotoxin adsorbent ^1^	1.50	1.50	1.50	1.50	1.50	1.50	1.50	1.50
Mineral premix ^2^	0.10	0.10	0.10	0.10	0.10	0.10	0.10	0.10
Vitamin premix ^3^	0.15	0.15	0.15	0.15	0.15	0.15	0.15	0.15
Calculated Values								
ME, kcal/kg	3350	3350	3350	3350	3325	3325	3325	3325
Crude protein, %	17.54	17.66	16.21	16.34	14.9	15	13.56	13.66
SID lysine, %	1.10	1.10	1.00	1.00	0.90	0.90	0.8	0.8
Calcium, %	0.70	0.54	0.65	0.49	0.60	0.44	0.55	0.39
Total phosphorus, %	0.56	0.38	0.51	0.33	0.47	0.29	0.43	0.27
Available phosphorus, %	0.36	0.18	0.31	0.13	0.28	0.1	0.25	0.09
Calcium: available phosphorus ratio	1.94	3.0	2.09	3.76	2.14	4.4	2.2	4.33
Sodium, %	0.20	0.20	0.20	0.200	0.18	0.18	0.17	0.17
SID Met + Cys/SID Lys	0.59	0.59	0.59	0.59	0.60	0.60	0.60	0.60
SID Tre/SID Lys	0.65	0.65	0.65	0.65	0.65	0.65	0.65	0.65
SID Trip/SID Lys	0.20	0.20	0.20	0.20	0.20	0.20	0.200	0.2
SID Val/SID Lys	0.69	0.69	0.69	0.69	0.69	0.69	0.69	0.69
SID Ile/SID Lys	0.55	0.55	0.55	0.55	0.55	0.55	0.55	0.55

PC: Positive Control; NC: Negative Control; ME: metabolizable energy; SID = standardized ileal digestibility; ^1^ Mycotoxin adsorbent; ^2^ Mineral premix provided per kg of diet: 100 mg Fe; 10 mg Cu; 40 mg Mn; 1 mg Co; 100 mg Zn; 1.5 mg I. ^3^ Vitamin premix provided per kg of diet: 6000 IU vitamin A; 1500 IU vitamin D_3_; 15 mg vitamin E; 1.5 mg vitamin K_3_; 1.35 mg vitamin B_1_; 4 mg vitamin B_2_; 2 mg vitamin B_6_; 20 μg vitamin B_12_; 20 mg niacin; 9.35 mg pantothenic acid; 600 μg folic acid; 80 μg biotin; 300 μg Se.

**Table 3:** Average initial weight (IW), average daily feed intake (ADFI), average daily gain (ADG), feed conversion ratio (FCR), and final weight (FW) of pigs during each phase of the nursery period according to the experimental treatments.

Parameters	Treatments					C.V. (%)	*p*-Value	*p*-Value		
	PC	NC	1000 FYT	2000 FYT	3000 FYT			Linear	Quadratic	Cubic
Pre-starter I (21–28 days)										
IW (kg)	6. 080	6.078	6.079	6.082	6.08	12.20	1.000	NS	NS	NS
ADFI (kg)	0.2	0.185	0.161	0.177	0.154	22.30	0.086	NS	NS	NS
ADG (kg)	0.17	0.145	0.133	0.143	0.122	31.80	0.226	NS	NS	NS
FCR	1.379	1.304	1.255	1.267	1.271	23.60	0.937	NS	NS	NS
FW (kg)	7.273	7.093	7.007	7.086	6.937	12.00	0.956	NS	NS	NS
Pre-starter II (29–35 days)										
ADFI (kg)	0.445	0.439	0.411	0.415	0.401	13.60	0.395	NS	NS	NS
ADG (kg)	0.34	0.306	0.323	0.323	0.313	13.70	0.462	NS	NS	NS
FCR	1.317 ab	1.435 a	1.274 b	1.289 b	1.286 b	9.30	0.035	NS	0.0146 ^a^	NS
FW (kg)	9.653	9.29	9.269	9.35	9.129	10.70	0.913	NS	NS	NS
Starter I (36–49 days)										
ADFI (kg)	0.751	0.701	0.674	0.668	0.66	11.90	0.087	NS	NS	NS
ADG (kg)	0.489 a	0.365 b	0.410 b	0.425 ab	0.420 ab	16.40	0.001	NS	0.0247 ^b^	NS
FCR	1.553 b	1.924 a	1.656 b	1.572 b	1.574 b	10.40	<0.001	NS	0.000 ^c^	NS
FW (kg)	16.592	14.40	15.010	15.294	15.004	10.90	0.085	NS	NS	NS
Starter II (50–63 days)										
ADFI (kg)	1.116	0.986	1.027	1.041	1.030	9.30	0.083	NS	NS	NS
ADG (kg)	0.698	0.602	0.652	0.664	0.646	11.60	0.051	NS	NS	NS
FCR	1.601	1.643	1.589	1.577	1.601	6.90	0.815	NS	NS	NS
FW (kg)	26.360 a	22.838 b	24.132 ab	24.587 ab	24.048 ab	10.10	0.023	NS	NS	NS
Total (21–63 days)										
ADFI (kg)	0.729	0.667	0.662	0.668	0.656	9.40	0.058	NS	NS	NS
ADG (kg)	0.481 a	0.398 b	0.430 ab	0.441 ab	0.428 ab	11.00	0.001	NS	NS	NS
FCR	1.519 b	1.676 a	1.546 b	1.517 b	1.535 b	5.50	0.001	NS	0.0176 ^d^	NS

PC = Positive Control; NC = Negative Control; 1000 FYT = NC + 1000 FYT/kg; 2000 FYT = NC + 2000 FYT/kg; 3000 FYT = NC + 3000 FYT/kg. FYT = phytase units per kg of feed. C.V. = coefficient of variation. NS = not significant. Means followed by different letters in the same row are significantly different by Tukey’s test at 5%. *p*-value = Linear and quadratic response to increasing levels of phytase in the diet. ^a^ Y = 1.4256 − 0.0002X + 0.00000009449X^2^; *p*-value = 0.0146; R^2^= 0.2447. ^b^ Y = 0.3659 + 0.00005.5135X − 0.000000012466X^2^; *p*-value = 0.0247; R^2^ = 0.1257. ^c^ Y = 1.9193 − 0.0003X + 0.000000067566X^2^; *p*-value = 0.0001; R^2^ = 0.05717. ^d^ Y = 1.6689 − 0.0001X + 0.000000035261X^2^; *p*-value = 0.0176; R^2^ = 0.5463.

**Table 4:** Means of initial weight (IW), average daily feed intake (ADFI), average daily gain (ADG), feed conversion ratio (FCR), and final weight (FW) of pigs during the growth and finishing phases according to the experimental treatments.

Parameters	Treatments					C.V. (%)	*p*-Value	*p*-Value		
	PC	NC	1000 FYT	2000 FYT	3000 FYT			Linear	Quadratic	Cubic
Growing I (64–91 days)										
IW (kg)	26.360 a	22.838 b	24.132 ab	24.587 ab	24.048 ab	10.10	0.023	NS	NS	NS
ADFI (kg)	1.922 a	1.590 b	1.737 ab	1.741 ab	1.779 ab	11.30	0.019	0.0236 ^c^	NS	NS
ADG (kg)	1.001 a	0.771 b	0.940 a	0.954 a	0.964 a	11.30	<0.001	NS	<0.001 ^b^	NS
FCR	1.918 b	2.060 a	1.846 b	1.829 b	1.844 b	7.50	<0.001	NS	0.0013 ^d^	NS
FW (kg)	54.393 a	44.434 b	50.672 a	51.303 a	51.052 a	10.00	<0.001	NS	0.0018 ^e^	NS
Growing II (92–112 days)										
ADFI (kg)	2.535 a	2.060 b	2.385 a	2.494 a	2.479 a	12.00	<0.001	NS	<0.001 ^g^	NS
ADG (kg)	1.060 a	0.826 b	1.012 a	1.056 a	1.059 a	12.20	<0.001	<0.001 ^f^	NS	NS
FCR	2.396	2.502	2.352	2.363	2.338	5.90	0.140	NS	0.109 ^h^	NS
FW (kg)	76.644 a	61.780 b	71.929 a	73.469 a	73.296 a	9.90	<0.001	NS	<0.001 ^i^	NS
Finishing I (113–133 days)										
ADFI (kg)	3.135 a	2.544 b	3.095 a	3.103 a	3.139 a	12.00	0.001	<0.001 ^k^	NS	NS
ADG (kg)	1.159 a	0.880 b	1.203 a	1.181 a	1.209 a	13.50	<0.001	NS	<0.001 ^j^	NS
FCR	2.707 ab	2.899 a	2.575 b	2.625 b	2.596 b	7.30	0.006	NS	0.0016 ^l^	NS
FW (kg)	100.977 a	80.261 b	97.187 a	98.278 a	99.156 a	10.00	<0.001	<0.001 ^m^	NS	NS
Finishing II (134–156 days)										
ADFI (kg)	3.107 a	2.491 b	2.981 a	2.985 a	3.105 a	13.00	0.031	<0.001 ^o^	NS	NS
ADG (kg)	0.961 a	0.685 b	0.932 a	0.949 a	0.981 a	15.70	0.001	<0.001 ^n^	NS	NS
FCR	3.237	3.698	3.203	3.149	3.172	10.70	0.054	NS	0.0013 ^p^	NS
FW (kg)	122.127 a	95.605 b	117.665 a	119.166 a	120.765 a	10.50	<0.001	<0.001 ^q^	NS	NS
Total (64–156 days)										
ADFI (kg)	2.623 a	2.130 b	2.491 a	2.521 a	2.566 a	10.80	0.003	<0.001 ^s^	NS	NS
ADG (kg)	1.041 a	0.788 b	1.014 a	1.028 a	1.046 a	11.80	<0.001	<0.001 ^r^	NS	NS
FCR	2.519 b	2.705 a	2.454 b	2.450 b	2.452 b	5.70	0.001	NS	<0.001 ^t^	NS

PC = Positive Control; NC = Negative Control; 1000 FYT = NC + 1000 FYT/kg; 2000 FYT = NC + 2000 FYT/kg; 3000 FYT = NC + 3000 FYT/kg. FYT = phytase units per kg of feed. C.V. = coefficient of variation. NS = not significant. Means followed by different letters in the same row are significantly different by Tukey’s test at 5%. *p*-value = Linear and quadratic response to increasing levels of phytase in the diet. ^b^ Y = 0.7788 + 0.0002X − 0.000000039614X^2^; *p*-value = 0.0000; R^2^ = 0.5667. ^c^ Y = 1.6257 + 0.000057266X; *p*-value = 0.0236; R^2^ = 0.1277. ^d^ Y = 2.0516 − 0.0002X + 0.000000057351X^2^; *p*-value = 0.0013; R^2^ = 0.3832. ^e^ Y = 44.6702 + 0.0069X − 0.0000016221X^2^; *p*-value = 0.0018; R^2^ = 0.3433. ^f^ Y = 0.8768 + 0.00074305X; *p*-value = 0.000; R^2^ = 0.4190. ^g^ Y = 2.0646 + 0.0004X − 0.000000085018X^2^; *p*-value = 0.0006; R^2^ = 0.3542. ^h^ Y = 2.4923 − 0.0001X + 0.000000031283X^2^; *p*-value = 0.109; R^2^ = 0.1587. ^i^ Y = 62.1243 + 0.0114X − 0.0000025806X^2^; *p*-value = 0.0001; R^2^ = 0.3351. ^j^ Y = 0.8997 + 0.0003X − 0.000000073695X^2^; *p*-value = 0.000; R^2^ = 0.4503. ^k^ Y = 2.7017 + 0.0002X; *p*-value = 0.0002; R^2^ = 0.0.3062. ^l^ Y = 2.876 − 0.0003X + 0.000000073773X^2^; *p*-value = 0.0016; R^2^ = 0.3707. ^m^ Y = 85.0541 + 0.0058X; *p*-value = 0.0000; R^2^ = 0.4438. ^n^ Y = 0.7506 + 0.000090654X; *p*-value = 0.000; R^2^ = 0.4629. ^o^ Y = 2.614 + 0.0002X; *p*-value *p* = 0.0003; R^2^ = 0.2902. ^p^ Y = 2.5216 + 0.0005X − 0.000000092402X^2^; *p*-value =0.0013; R^2^ = 0.3618. ^q^ Y = 101.753 + 0.0077X; *p*-value = 0.000; R^2^ = 0.4819. ^r^ Y = 0.8508 + 0.000078769X; *p*-value = 0.000; R^2^ = 0.5278. ^s^ Y = 2.2262 + 0.0001X; *p*-value = 0.0001; R^2^ = 0.3256. ^t^ Y = 2.6931 − 0.0003X + 0.000000063136X^2^; *p*-value = 0.0001; R^2^= 0.5169.

**Table 5:** Average daily feed intake (ADFI), average daily gain (ADG), and feed conversion ratio (FCR) of pigs over the entire experimental period (21 to 156 days) according to the experimental treatments.

Parameters	Treatments					C.V. (%)	*p*-Value	*p*-Value		
	PC	NC	1000 FYT	2000 FYT	3000 FYT			Linear	Quadratic	Cubic
ADFI (kg)	0.867 a	0.667 b	0.832 a	0.845 a	0.854 a	10.9	<0.001	<0.001 ^b^	NS	NS
ADG (kg)	2.034 a	1.674 b	1.922 a	1.945 a	1.972 a	10.1	<0.001	<0.001 ^c^	NS	NS
FCR	2.346 b	2.512 a	2.308 b	2.299 b	2.309 b	5	<0.001	NS	<0.001 ^d^	NS

PC = Positive Control; NC = Negative Control; 1000 FYT = NC + 1000 FYT/kg; 2000 FYT = NC + 2000 FYT/kg; 3000 FYT = NC + 3000 FYT/kg. FYT = phytase units per kg of feed. C.V. = coefficient of variation. NS = not significant. Means followed by different letters in the same row are significantly different by Tukey’s test at 5%. *p*-value = Linear and quadratic response to increasing levels of phytase in the diet. ^b^ Y = 0.7134 + 0.000057401X; *p*-value = 0.0000; R^2^ = 0.6999. ^c^ Y = 1.7411 + 0.000091474X; *p*-value = 0.0003; R^2^ = 0.5412. ^d^ Y = 2.5033 − 0.0002X + 0.000000053575X^2^; *p*-value = 0.0001; R^2^ = 0.5753.

**Table 6:** Mean final weight (FW), carcass weight (CW), carcass yield (CY), backfat thickness (BT), loin depth (LD), percentage of lean meat (PLM), and lean meat content (LM, kg) of pigs fed diets with different phytase inclusion levels.

Parameters	Treatments					C.V. (%)	*p*-Value	*p*-Value		
	PC	NC	1000 FYT	2000 FYT	3000 FYT			Linear	Quadratic	Cubic
FW (kg)	122.020 a	101.323 b	117.513 a	118.863 a	122.107 a	9.6	<0.001	<0.001 ^b^	NS	NS
CW (kg)	87.031 a	69.411 b	82.823 a	84.449 a	86.907 a	10.3	<0.001	<0.001 ^c^	NS	NS
CY (%)	71.377 a	68.619 b	70.473 a	71.058 a	71.232 a	3.8	0.0370	<0.001 ^d^	NS	NS
BT (mm)	16.940	16.701	16.313	16.617	16.555	21.6	0.9370	NS	NS	NS
LD (mm)	64.180 a	57.585 b	62.598 a	63.605 a	62.829 a	11.7	0.0210	NS	0.0031 ^e^	NS
PLM (%)	56.061 a	53.662 b	55.792 ab	55.853 a	55.874 a	4.4	0.0200	0.0420 ^f^	NS	NS
LM (kg)	48.253 a	37.969 b	45.983 a	47.057 a	48.376 a	10.4	<0.001	<0.001 ^g^	NS	NS

PC = Positive Control; NC = Negative Control; 1000 FYT = NC + 1000 FYT/kg; 2000 FYT = NC + 2000 FYT/kg; 3000 FYT = NC + 3000 FYT/kg. FYT = phytase units per kg of feed. C.V. = coefficient of variation. NS = not significant. Means followed by different letters in the same row are significantly different by Tukey’s test at 5%. *p*-value = Linear and quadratic response to increasing levels of phytase in the diet. ^b^ Y = 110.4871 + 0.0041X; *p*-value = 0.000; R^2^ = 0.162. ^c^ Y =76.0183 + 0.0039X; *p*-value = 0.000; R^2^ = 0.2406. ^d^ Y = 68.7987 + 0.001X; *p*-value = 0.009; R^2^ = 0.0927. ^e^ Y = 54.5703 + 0.008X − 0.00000182X^2^; *p*-value = 0.031; R^2^ = 0.074. ^f^ Y = 54.8014 + 0.0005X; *p*-value = 0.420; R^2^ = 0.0358. ^g^ Y = 41.6589 + 0.0025X; *p*-value = 0.000; R^2^ = 0.266.

**Table 7:** Mean values of lipid oxidation on day 0 (TBARS D0) and day 7 (TBARS D7), thawing loss (DLL), cooking loss (CL), marbling score, pH, pressure loss (PL), lightness (L), redness (a), and yellowness (b) according to the experimental treatments.

Parameters	Treatments					C.V. (%)	*p*-Value	*p*-Value		
	PC	NC	1000 FYT	2000 FYT	3000 FYT			Linear	Quadratic	Cubic
TBARS D0	0.09	0.09	0.089	0.093	0.087	9.6	0.8053	NS	NS	NS
DLL (%)	8.553	9.892	8.536	10.964	9.365	24	0.2407	NS	NS	NS
CL (%)	23.716	26.908	27.106	29.388	28.125	17.8	0.2485	NS	NS	NS
pH	5.847	5.719	5.815	5.779	5.709	4	0.7936	NS	NS	NS
Marbling	1.778	2.278	1.625	1.778	1.944	38.3	0.4763	NS	NS	NS
PL (%)	27.392	29.254	30.105	28.542	29.02	12.3	0.5326	NS	NS	NS
L	46.507	46.97	47.758	48.096	48.407	7.3	0.7093	NS	NS	NS
a	3.919	4.83	4.304	3.948	4.367	34.7	0.8368	NS	NS	NS
b	12.748	12.881	12.938	12.815	13.476	11.2	0.9213	NS	NS	NS
TBARS D7	0.096	0.073	0.09	0.095	0.089	35.9	0.4433	NS	NS	NS

PC = Positive Control; NC = Negative Control; 1000 FYT = NC + 1000 FYT/kg; 2000 FYT = NC + 2000 FYT/kg; 3000 FYT = NC + 3000 FYT/kg. FYT = phytase units per kg of feed. C.V. = coefficient of variation. NS = not significant. *p*-value = Linear and quadratic response to increasing levels of phytase in the diet.

## Data Availability

The original contributions presented in this study are included in the article/Supplementary Materials. Further inquiries can be directed to the corresponding author.

## Supplementary Materials

The following supporting information can be downloaded at: https://www.mdpi.com/article/10.3390/vetsci13060516/s1, Supplementary Figure S1. Regression-based dose–response effects of phytase supplementation on selected nursery performance responses. Supplementary Figure S2. Regression-based dose–response effects of phytase supplementation on performance responses during Growing I. Supplementary Figure S3. Regression-based dose–response effects of phytase supplementation on performance responses during Growing II. Supplementary Figure S4. Regression-based dose–response effects of phytase supplementation on performance responses during Finishing I. Supplementary Figure S5. Regression-based dose–response effects of phytase supplementation on performance responses during Finishing II. Supplementary Figure S6. Regression-based dose–response effects of phytase supplementation on performance responses during the combined growing-finishing period. Supplementary Figure S7. Regression-based dose–response effects of phytase supplementation on total-period performance of pigs. Supplementary Figure S8. Regression-based dose–response effects of phytase supplementation on final weight, carcass weight, and carcass yield. Supplementary Figure S9. Regression-based dose–response effects of phytase supplementation on loin depth and carcass lean meat deposition.
