# Supplementary material for: Phytase Overdose in Diets for Pigs from Weaning to Slaughter: Effects on Performance, Carcass and Meat Quality
Source: Vet Sci. 2026 May 26;13(6):516. doi: 10.3390/vetsci13060516 (PMC13307599; doi:10.3390/vetsci13060516)
Supplement: Supplementary file 1 [file vetsci-13-00516-s001.zip › vetsci-4320427-supplementary.pdf]

Supplementary Figure S1. Regression-based dose–response effects of phytase supplementation on selected nursery performance responses.

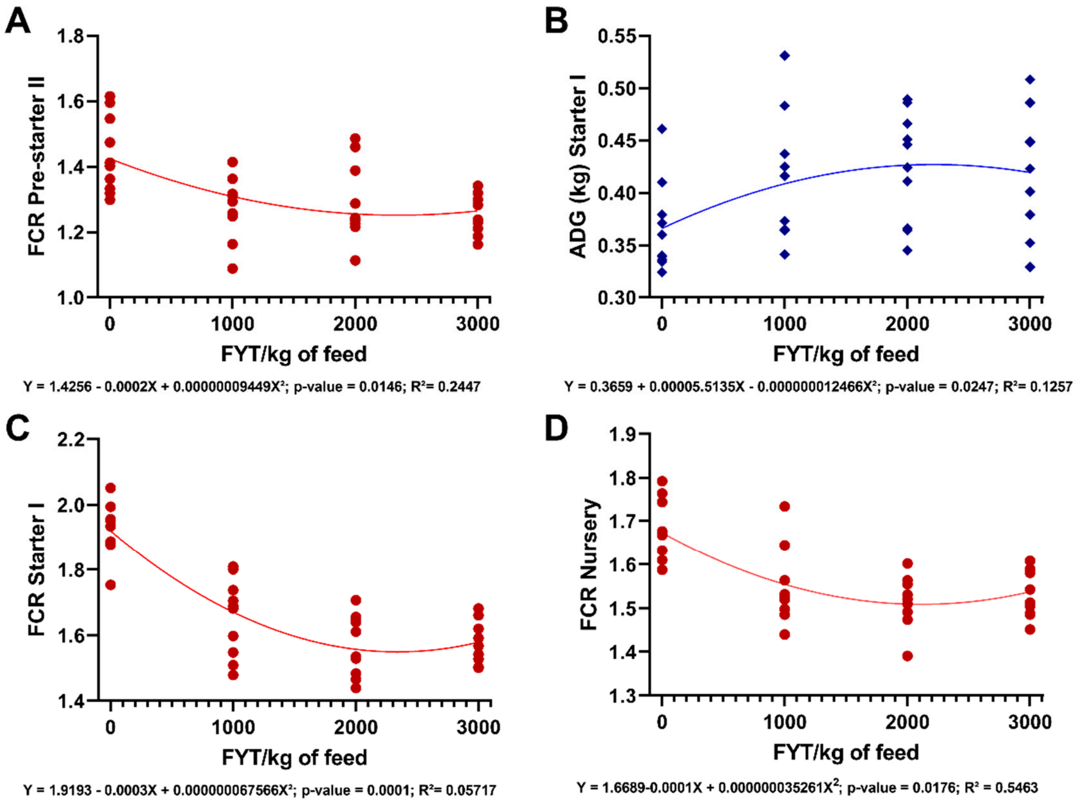

Caption/footnote: Dose–response regressions for selected performance variables in nursery pigs fed diets supplemented with increasing phytase levels. Panels show: **(A)** feed conversion ratio during Pre-starter II, **(B)** average daily gain during Starter I, **(C)** feed conversion ratio during Starter I, and **(D)** feed conversion ratio during the overall nursery period. Symbols represent experimental observations, and fitted curves indicate the respective quadratic regression models displayed within each panel. Phytase supplementation levels are expressed as FYT/kg of feed. **FCR** = feed conversion ratio; **ADG** = average daily gain; **FYT** = phytase units; **R^2** = coefficient of determination.

Supplementary Figure S2. Regression-based dose–response effects of phytase supplementation on performance responses during Growing I.

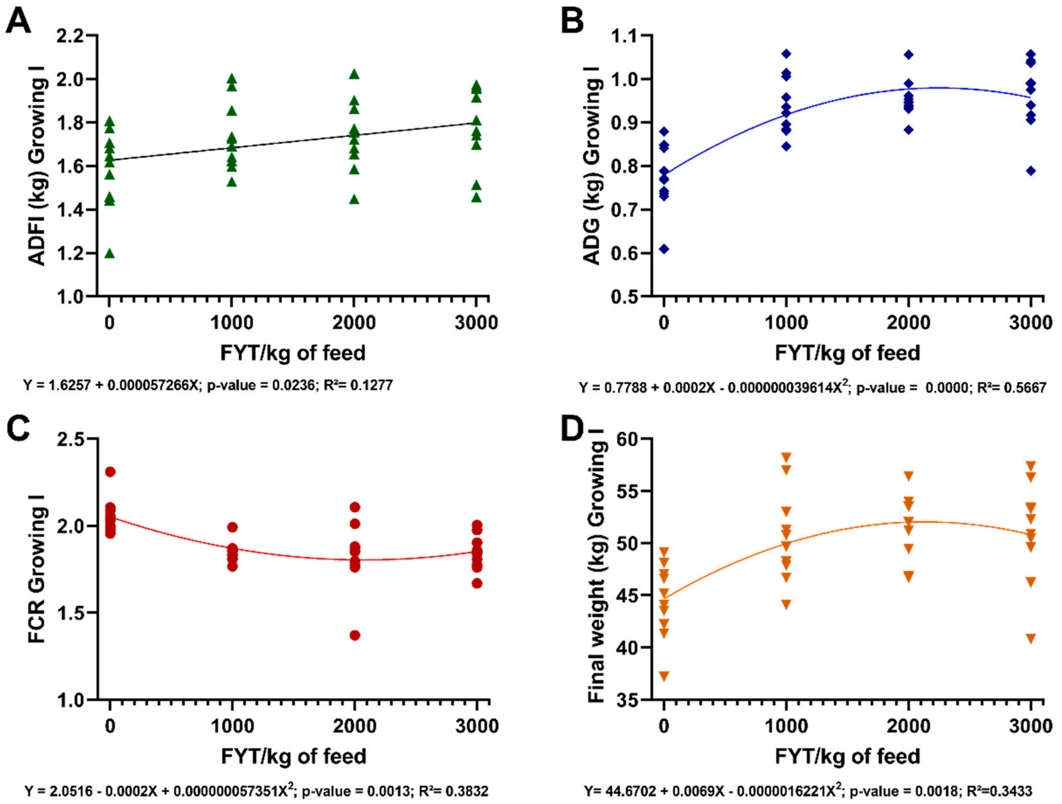

Caption/footnote:

Dose–response regressions for productive performance variables in pigs during Growing I fed diets supplemented with increasing phytase levels. Panels show: **(A)** average daily feed intake, **(B)** average daily gain, **(C)** feed conversion ratio, and **(D)** final weight. Symbols represent experimental observations, and fitted lines or curves indicate the respective regression models displayed within each panel. Phytase supplementation levels are expressed as FYT/kg of feed. **ADFI** = average daily feed intake; **ADG** = average daily gain; **FCR** = feed conversion ratio; **FW** = final weight; **FYT** = phytase units; $\mathbf{R^2}$ = coefficient of determination.

Supplementary Figure S3. Regression-based dose–response effects of phytase supplementation on performance responses during Growing II.

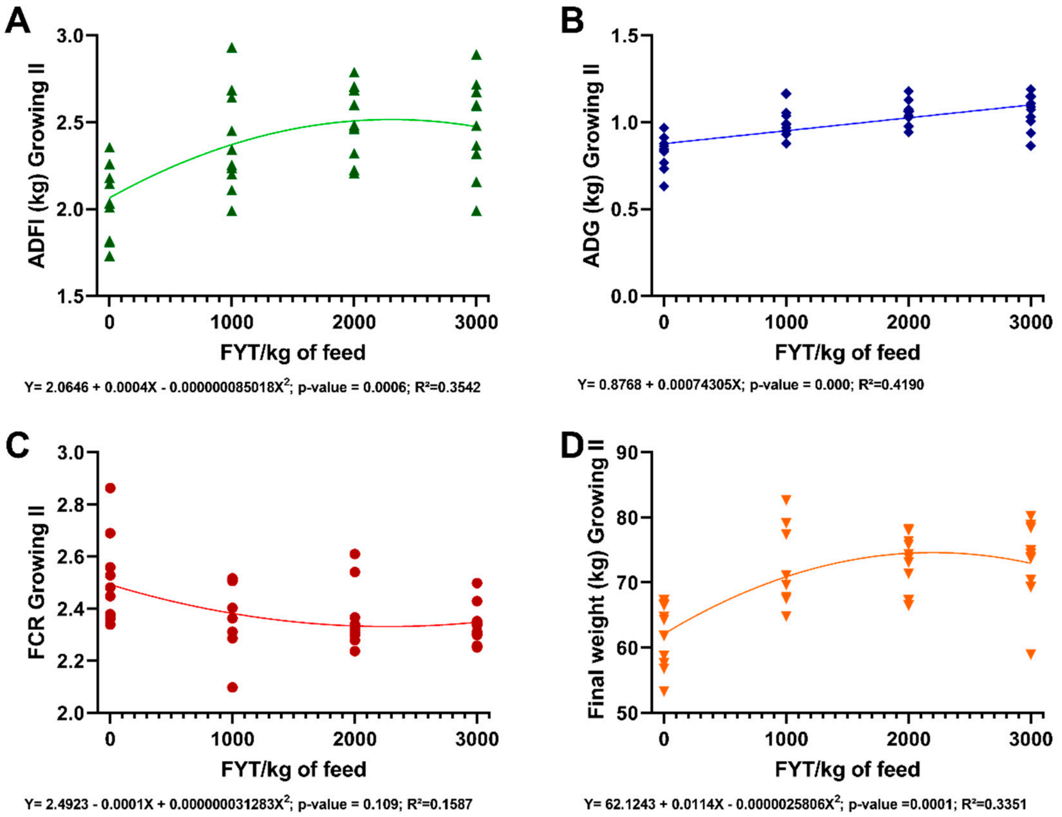

Caption/footnote:

Dose–response regressions for productive performance variables in pigs during Growing II fed diets supplemented with increasing phytase levels. Panels show: **(A)** average daily feed intake, **(B)** average daily gain, **(C)** feed conversion ratio, and **(D)** final weight. Symbols represent experimental observations, and fitted lines or curves indicate the respective regression models displayed within each panel. Phytase supplementation levels are expressed as FYT/kg of feed. **ADFI** = average daily feed intake; **ADG** = average daily gain; **FCR** = feed conversion ratio; **FW** = final weight; **FYT** = phytase units; **R^2** = coefficient of determination.

Supplementary Figure S4. Regression-based dose–response effects of phytase supplementation on performance responses during Finishing I.

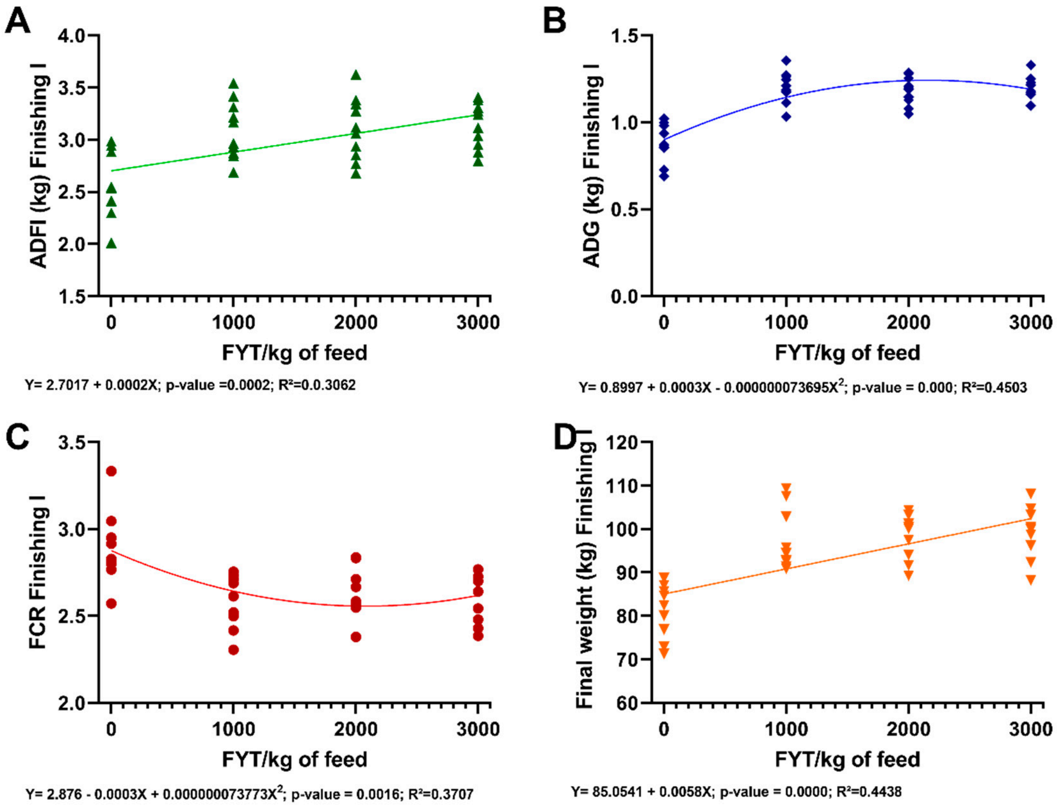

Caption/footnote:

Dose–response regressions for productive performance variables in pigs during Finishing I fed diets supplemented with increasing phytase levels. Panels show: **(A)** average daily feed intake, **(B)** average daily gain, **(C)** feed conversion ratio, and **(D)** final weight. Symbols represent experimental observations, and fitted lines or curves indicate the respective regression models displayed within each panel. Phytase supplementation levels are expressed as FYT/kg of feed. **ADFI** = average daily feed intake; **ADG** = average daily gain; **FCR** = feed conversion ratio; **FW** = final weight; **FYT** = phytase units; **R^2** = coefficient of determination.

Supplementary Figure S5. Regression-based dose–response effects of phytase supplementation on performance responses during Finishing II.

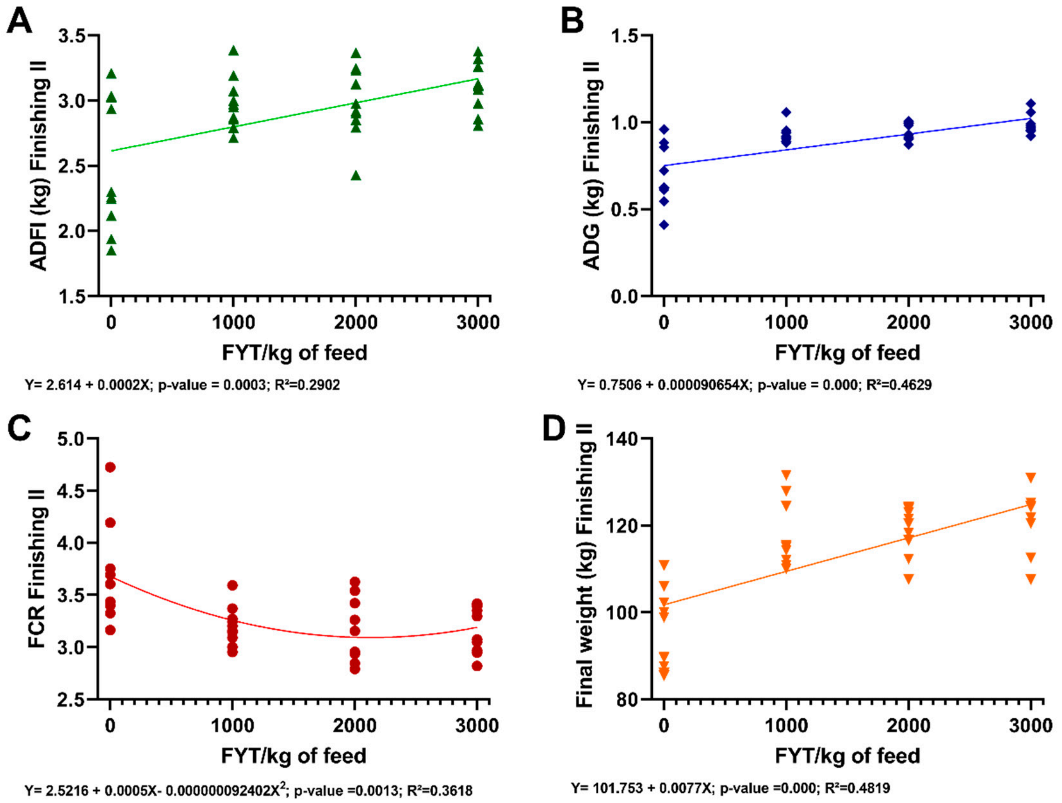

Caption/footnote:

Dose–response regressions for productive performance variables in pigs during Finishing II fed diets supplemented with increasing phytase levels. Panels show: **(A)** average daily feed intake, **(B)** average daily gain, **(C)** feed conversion ratio, and **(D)** final weight. Symbols represent experimental observations, and fitted lines or curves indicate the respective regression models displayed within each panel. Phytase supplementation levels are expressed as FYT/kg of feed. **ADFI** = average daily feed intake; **ADG** = average daily gain; **FCR** = feed conversion ratio; **FW** = final weight; **FYT** = phytase units; **R^2** = coefficient of determination.

Supplementary Figure S6. Regression-based dose–response effects of phytase supplementation on performance responses during the combined growing-finishing period.

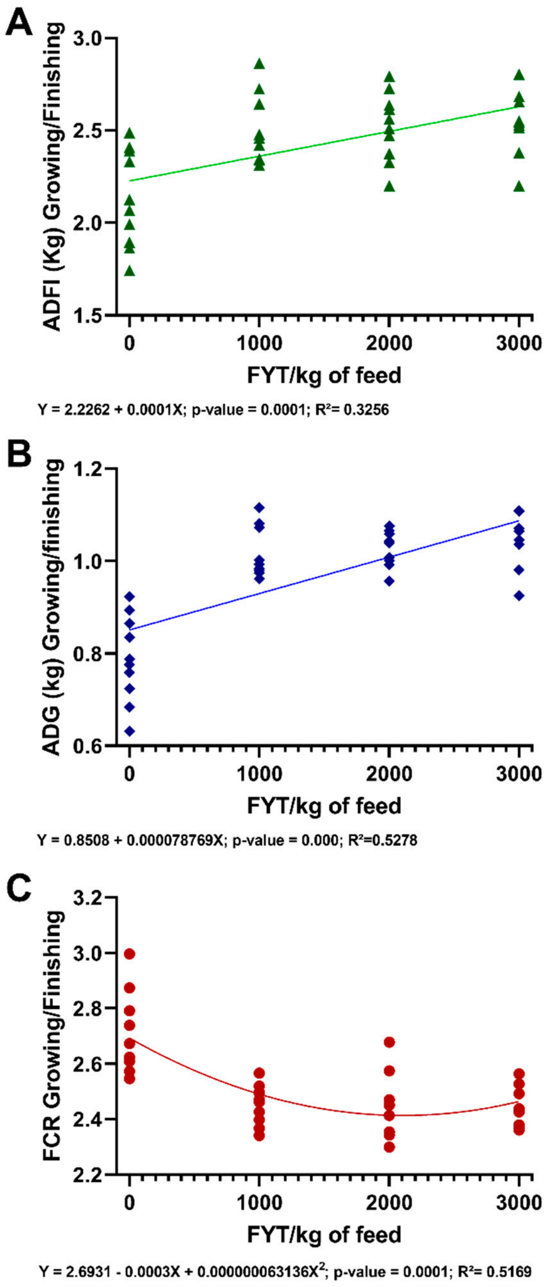

Caption/footnote:

Dose–response regressions for productive performance variables in pigs fed diets supplemented with increasing phytase levels during the combined growing-finishing period. Panels show: **(A)** average daily feed intake, **(B)** average daily gain, and **(C)** feed conversion ratio. Symbols represent experimental observations, and fitted lines or curves indicate the respective regression models displayed within each panel. Phytase supplementation levels are expressed as FYT/kg of feed. **ADFI** = average daily feed intake; **ADG** = average daily gain; **FCR** = feed conversion ratio; **FYT** = phytase units; **R^2** = coefficient of determination.

Supplementary Figure S7. Regression-based dose–response effects of phytase supplementation on total-period performance of pigs.

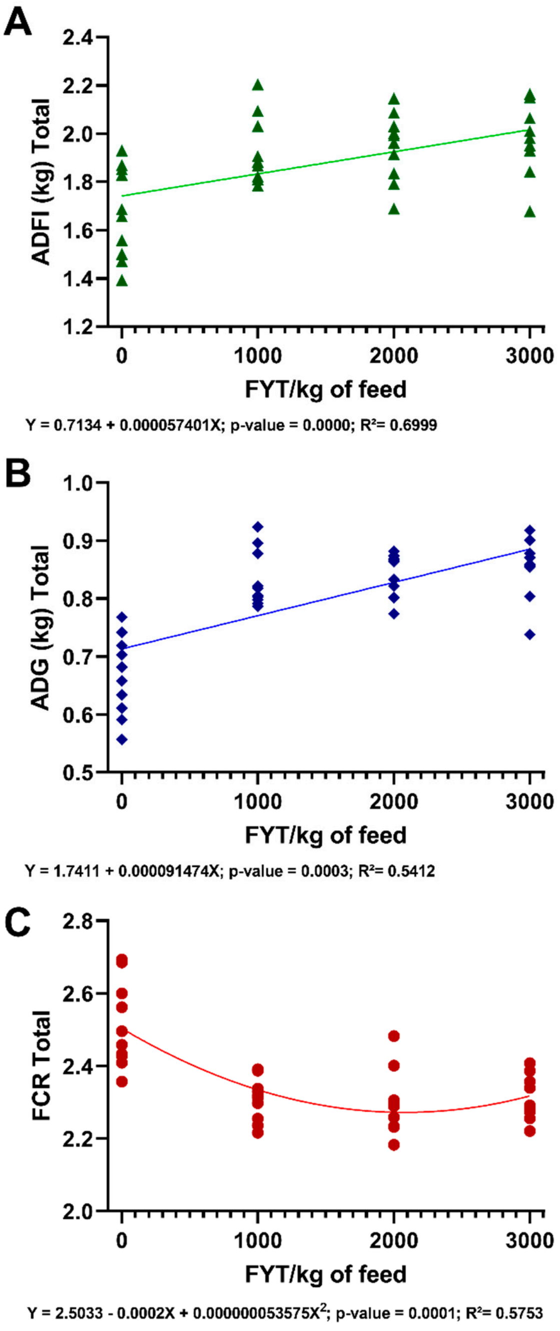

Caption/footnote:

Dose–response regressions for overall performance variables in pigs receiving increasing dietary phytase supplementation levels during the complete experimental period. Panels show: **(A)** average daily feed intake, **(B)** average daily gain, and **(C)** feed conversion ratio. Symbols represent experimental observations, and fitted lines or curves indicate the respective regression models displayed within each panel. Phytase supplementation levels are expressed as FYT/kg of feed. **ADFI** = average daily feed intake; **ADG** = average daily gain; **FCR** = feed conversion ratio; **FYT** = phytase units; **R^2** = coefficient of determination.

Supplementary Figure S8. Regression-based dose–response effects of phytase supplementation on final weight, carcass weight, and carcass yield.

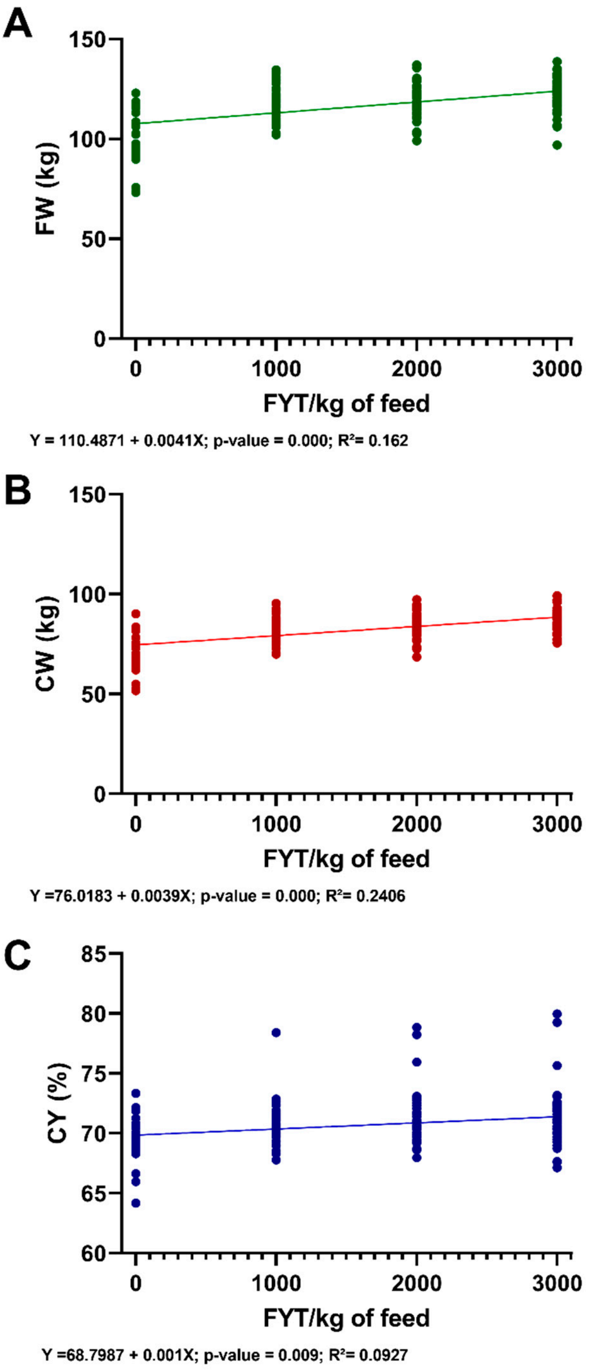

Caption/footnote:

Dose–response regressions for final weight and carcass yield-related variables in pigs fed diets supplemented with increasing phytase levels. Panels show: **(A)** final weight, **(B)** carcass weight, and **(C)** carcass yield. Symbols represent experimental observations, and fitted lines indicate the respective linear regression models displayed within each panel. Phytase supplementation levels are expressed as FYT/kg of feed. **FW** = final weight; **CW** = carcass weight; **CY** = carcass yield; **FYT** = phytase units; **R^2** = coefficient of determination.

Supplementary Figure S9. Regression-based dose–response effects of phytase supplementation on loin depth and carcass lean meat deposition.

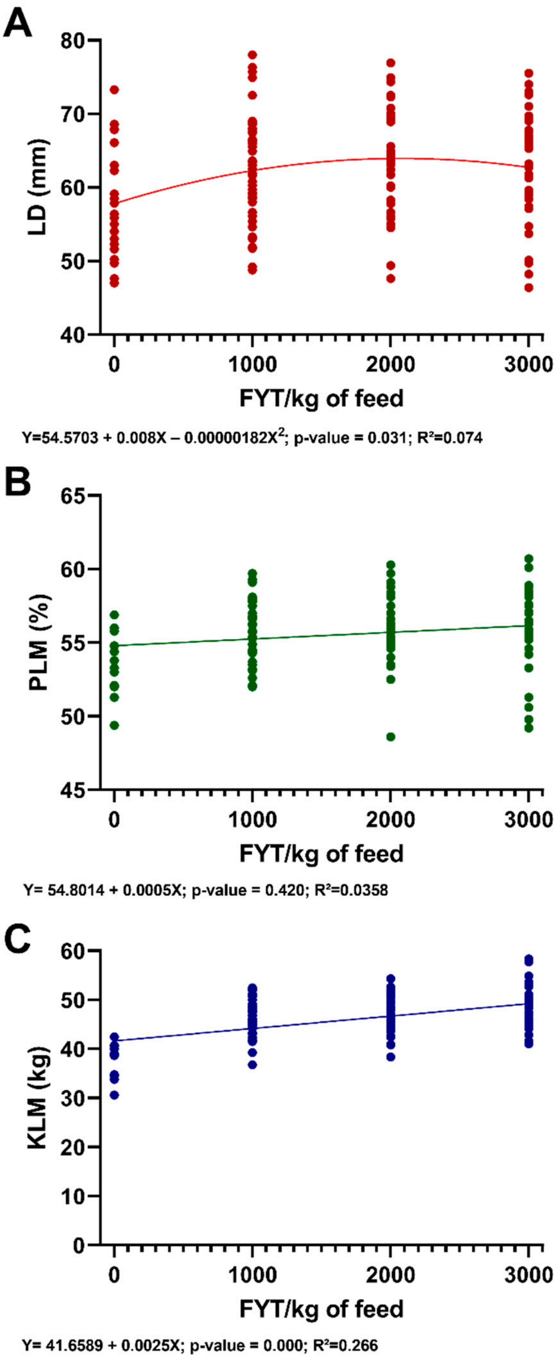

Caption/footnote:

Dose–response regressions for loin depth and carcass lean meat deposition in pigs fed diets supplemented with increasing phytase levels. Panels show: **(A)** loin depth, **(B)** percentage of lean meat, and **(C)** kilograms of lean meat. Symbols represent experimental observations, and fitted lines or curves indicate the respective regression models displayed within each panel. Phytase supplementation levels are expressed as FYT/kg of feed. **LD** = loin depth; **PLM** = percentage of lean meat; **LM** = kilograms of lean meat; **FYT** = phytase units; **R^2** = coefficient of determination.
